# Multilevel Proteomic Profiling of Colorectal Adenocarcinoma Caco-2 Cell Differentiation to Characterize an Intestinal Epithelial Model

**DOI:** 10.1021/acs.jproteome.4c00276

**Published:** 2024-05-29

**Authors:** Emily Ef Fekete, Angela Wang, Marybeth Creskey, Sarah E Cummings, Jessie R Lavoie, Zhibin Ning, Jianjun Li, Daniel Figeys, Rui Chen, Xu Zhang

**Affiliations:** † Regulatory Research Division, Biologic and Radiopharmaceutical Drugs Directorate, Health Products and Food Branch, Health Canada, Ottawa K1A 0K9, Canada; ‡ Department of Biochemistry, Microbiology and Immunology, Faculty of Medicine, University of Ottawa, Ottawa K1H8M5, Canada; § School of Pharmaceutical Sciences, Faculty of Medicine, University of Ottawa, Ottawa K1H8M5, Canada; ∥ Human Health Therapeutics Research Centre, National Research Council Canada, Ottawa, Ontario K1A0R6, Canada

**Keywords:** Caco-2, differentiation, intestinal cellular model, microbiome, proteomics

## Abstract

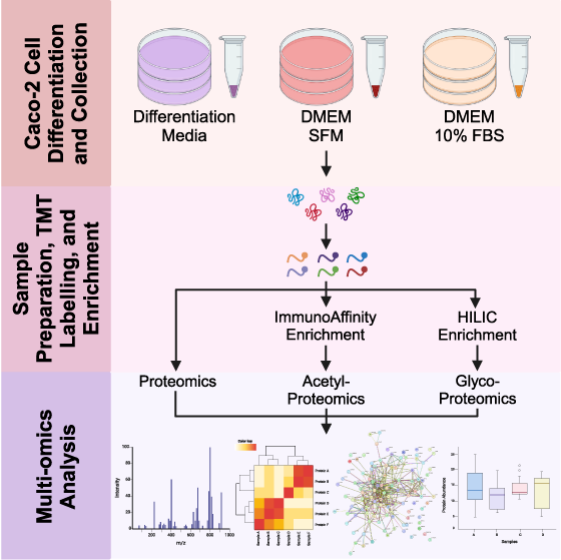

Emergent advancements on the role of the intestinal microbiome for human health and disease necessitate well-defined intestinal cellular models to study and rapidly assess host, microbiome, and drug interactions. Differentiated Caco-2 cell line is commonly utilized as an epithelial model for drug permeability studies and has more recently been utilized for investigating host–microbiome interactions. However, its suitability to study such interactions remains to be characterized. Here, we employed multilevel proteomics to demonstrate that both spontaneous and butyrate-induced Caco-2 differentiations displayed similar protein and pathway changes, including the downregulation of proteins related to translation and proliferation and upregulation of functions implicated in host–microbiome interactions, such as cell adhesion, tight junction, extracellular vesicles, and responses to stimuli. Lysine acetylomics revealed that histone protein acetylation levels were decreased along with cell differentiation, while the acetylation in proteins associated with mitochondrial functions was increased. This study also demonstrates that, compared to spontaneous differentiation methods, butyrate-containing medium accelerates Caco-2 differentiation, with earlier upregulation of proteins related to host–microbiome interactions, suggesting its superiority for assay development using this intestinal model. Altogether, this multiomics study emphasizes the controlled progression of Caco-2 differentiation toward a specialized intestinal epithelial-like cell and establishes its suitability for investigating the host–microbiome interactions.

## Introduction

The gastrointestinal (GI) tract is an important collection of organs in the human body, which is involved in various biological processes, including nutrition and immune development.^1^ Diseases originating from the GI tract, such as colorectal cancer and inflammatory bowel diseases (IBD), are among the most common diseases in modern society.^1,2^ The GI tract is also the home of diverse human symbiotic microbial communities, namely the human microbiome.^1^ The gut microbiome in particular has gained considerable attention in recent years due to its profound impact on human health and disease.^1,2^ Increasingly, causative roles of gut microbiota in diseases have been identified, leading to the intensive development and application of microbiota-directed therapeutics, such as the use of fecal microbiota transplantation (FMT) for preventing recurrent *C. difficile* infection.^3 −5^ The establishment of appropriate *in vitro* models that accurately recapitulate host–microbiome interactions is crucial for advancing our understanding of the complex interplay between the human body and its resident microbial communities, as well as the efficient risk and quality assessment of therapeutics targeting or derived from microbiomes.^3^

The intestinal epithelial barrier plays a vital role in host–microbiome interactions.^1^ Caco-2 cells, derived from human colon adenocarcinoma, have been widely employed as a prominent epithelial model for drug and intestinal disease studies due to their ability to mimic various aspects of intestinal physiology and provide insights into pathology of the intestinal epithelium.^6,7^ Caco-2 cells have demonstrated the ability to undergo differentiation into epithelium-like cells through both spontaneous and butyrate-induced processes.^6,8 −12^ Spontaneous differentiation of Caco-2 cells is primarily driven by the intrinsic program of the cells themselves. Once reaching confluence and establishing contact inhibition, cell-to-cell contact and communication generates a physical cue for the cells to differentiate, taking 21 days to reach full differentiation.^6,9,12^ The addition of butyrate, a metabolite derived from the microbial fermentation of dietary fiber, has been shown to promote intestinal epithelial cell differentiation, shortening the differentiation time needed to 7 days.^8,12^ In both cases, there is a progression away from a proliferative state toward a more differentiated and specialized phenotype over the course of one to a few weeks depending on differentiation methods used.^6,8,9,12^

To enable the application of the Caco-2 cellular model in microbiome drug development and host–microbiome interaction studies, there is a need to revisit and comprehensively characterize the molecular processes at play during differentiation. Previous studies have examined transcriptomic, proteomic, and glycomic alterations that Caco-2 cells undergo upon either spontaneous or butyrate-induced differentiation.^9,10,12^ In this study, we took advantage of tandem mass tag (TMT) multiplexing and enrichment strategies to further these previous efforts for in-depth, proteome-wide, and multilevel characterizations of the Caco-2 differentiation over a time course, including quantitative proteomics, lysine acetylproteomics, and glycoproteomics.^13^ We compared three commonly used Caco-2 differentiation protocols in the literature, including both spontaneous differentiations using serum-free (SFM) or serum-containing (DFBS) media and butyrate-containing medium-induced differentiation (DM). The observations in this study provide greater insights into the application of the Caco-2 cellular model for the study of host–microbiome interactions and the *in vitro* assay development for the assessment of microbiome-directed therapeutics.

## Materials and Methods

### Caco-2 Cell Culture and Differentiation

Human colorectal adenocarcinoma (Caco-2) cells were obtained from the American Type Culture Collection (ATCC, P/N HTB-37) and routinely maintained in Dulbecco’s modified Eagle’s medium (DMEM) with 10% heat-inactivated (HI) FBS (Gibco, P/N 12 484–028, lot: 2330105RP) at 37 °C in a humidified 5% CO_2_ incubator. Cells from passages 30–48 were used in this study. Cells were all confirmed to be mycoplasma negative using a PCR mycoplasma detection kit (Thermo Fisher Chemicals, P/N J66117) following manufacturer’s instructions.

For differentiation, Caco-2 cells were seeded (day 0) at a density of ∼1e^6^cells/cm^2^ in DMEM with 10% HI-FBS. On day 1, cells were switched into either serum-free DMEM (SFM) or DMEM with 10% HI-FBS (DFBS) or Corning Intestinal Epithelium Differentiation Medium (Corning, P/N 355357, DMEM containing sodium butyrate, and no serum extender supplement was added; DM). The Caco-2 cells were then cultured for 21 days in one of the three growth/differentiation medias, with media changes performed every other day. Cell imaging was performed using EVOS cell imaging system (Thermo Fisher Scientific). Cells were harvested on day 1, 3, 7, 14, and 21. Cells were harvested by removing culture medium, washing adherent cells twice with Dulbecco’s phosphate-buffered saline (DPBS), and lifted with 0.25% Trypsin-EDTA. To inactivate the trypsin, medium with 10% HI-FBS was added at a ratio of 1:1, and the cells were then pelleted at 400g for 5 min. Supernatant was discarded, and the cell pellet was washed with DPBS twice pelleting cells again at 400g for 5 min. The supernatant was removed, and the cell pellets were stored at −20 °C until processing.

### Protein Extraction, Digestion, and TMT Labeling

The cells were lysed by resuspending frozen cell pellets in 200 μL of lysis buffer (1% SDS in 100 mM TEAB) and sonicating them with a QSonica Q700 water-chilled cup-horn sonicator at 50% amplitude, 10 s pulse on/off cycle, for 10 min active sonication time at 8 °C. The lysate was then centrifuged at 16,000g for 10 min at 4 °C. Protein-containing supernatant was transferred to a new tube, and protein concentrations were determined using the Pierce BCA Protein Assay Kit (Thermo Fisher Scientific, P/N 23225) following the manufacturer protocol. 150 μg of protein lysates of each sample was reduced with 15 mM tris(2-carboxyethyl) phosphine (TCEP) at 55 °C for 1 h and alkylated with 25 mM iodoacetamide for 30 min at 21 °C protected from light. Proteins were then precipitated by adding 6 vol of ice-cold acetone at −20 °C overnight. Samples were then centrifuged at 14,000g for 10 min at 4 °C, and the acetone was carefully decanted without dislodging the protein pellet. The pellet was then air-dried for 5–10 min, resuspended with 75 μL of 100 mM TEAB containing 3 mAU of lysyl endopeptidase (1 mAU LysC: 50 μg protein), and incubated at 37 °C in a thermomixer at 500 rpm in the dark for 3 h. 75 μL of 100 mM TEAB containing 3 μg of MS-grade trypsin (1 μg trypsin: 50 μg protein) was then added and incubated overnight at 37 °C in a thermomixer. The digestion was stopped by acidifying each sample with 35 μL of 10% formic acid, and the digests were then dried on a centrivap for further TMT labeling.

TMT labeling was performed according to our previously established dry TMT labeling workflow using an 11-plex TMT Mass Tagging Kit (Thermo Fisher Scientific, P/N 90406 + A34807, lot XB323735).^13^ Briefly, dried peptide (150 μg) was resuspended in 37.5 μL of 100 mM TEAB/20% ACN, and 50 μg of each sample was combined to generate a pool sample. The remaining 100 μg peptides of each sample was added to 200 μg of lyophilized TMT reagents.^13^ Samples were randomized into 6 TMT 11-plex experiments with the first and 11th channels of each experiment as reference channels. The TMT reaction was performed by incubation at 25 °C for 2 h with shaking at 500 rpm and quenched with 4 μL of 5% hydroxylamine for 15 min at room temperature. The 11 samples of each TMT experiment were then combined (1100 μg per mixture) and dried with a centrivap. 100 μg of the mixture was fractionated using a high pH reverse phase fractionation kit (Thermo Fisher Scientific, P/N 84868) for proteomics, and the remaining 1 mg peptides of each mixture was used for lysine acetyl-peptide and glycopeptide enrichment.

### Immunoaffinity Enrichment of Lysine-Acetylated Peptides

The enrichment of lysine-acetylated peptides was performed using a PTMScan HS Acetyl-Lysine Motif [Ac–K] Kit (Cell Signaling Technology, P/N 50071) following the manufacturer’s instructions. Briefly, approximately 1 mg of TMT-labeled peptides of each mixture was resuspended in 1.5 mL of HS IAP bind buffer added to PBS-washed magnetic Ac–K motif-antibody beads and incubated on a rotator for 2 h at 4 °C for peptide binding. Unbound peptides were then washed with IAP wash buffer and water, and the Kac peptides were then eluted with 50 μL of 0.15% TFA twice. Both elutes were combined and dried with a centrivap, followed by desalting using Pierce C18 spin tips (P/N 84850). For desalting, the C18 columns were activated by washing three times with 50 μL of 100% acetonitrile (ACN) and equilibrated with 50 μL of 0.1% formic acid (FA); the sample was then resuspended in 0.1% FA, added to the column, and followed by two more washes with 0.1% FA and elution with 80%ACN/0.1%FA. The eluted peptide was then dried using a centrivap prior to LC-MSMS analysis.

### Hydrophilic Interaction Liquid Interaction Chromatography (HILIC) for Glycopeptides

Hydrophilic interaction solid phase extraction (HILIC SPE) was conducted as previously described with minor changes.^14^ TMT-labeled peptides of each mixture were solubilized in 50 μL of 80% acetonitrile (ACN) with 1% of trifluoroacetic acid (TFA) and loaded onto the microcolumn packed with 5 mg of polyhydroxylethyl beads (100 Å, 5 μm, PolyLC, Columbus, MA). After three washes with 50 μL of the same loading buffer, glycopeptides retained on SPE column were eluted by 50 μL 30% of ACN with 0.1% TFA. Eluted glycopeptides were lyophilized and then deglycosylated by incubation with 50 μL of 1 unit per microliter of PGNase F (New England Biolabs, Ipswich, MA) in 50 mM Tris-HCl (pH = 7.5) overnight at 37 °C. Deglycosylated peptides were dried with Speed Vac, and the samples were stored at −80 °C until MS analysis.

### HPLC-MS/MS Analysis

Unenriched and fractionated peptides were analyzed using an Orbitrap Fusion Lumos mass spectrometer (Thermo Fisher Scientific) coupled to an Easy-nLC 1200 system for UPLC (Thermo Fisher Scientific). The instrument was calibrated by infusion prior to analysis with Pierce FlexMix Calibration Solution (P/N A39239). For each fraction, 20 μL of 0.1% FA was used to resuspend peptides, and 2 μL was analyzed by loading onto a NanoViper Acclaim pepmap 100 trap column (75 μm diameter, 20 mm length with 3 μm C18 beads) and desalting with 0.1% formic acid in water (solvent A), before separating on an NanoViper Acclaim pepmap C18 reverse-phase analytical column (50 μm diameter, 150 mm length with 2 μm C18 beads). Chromatographic separation was achieved at a flow rate of 0.300 μL/min—over 100 min in five linear steps as follows (solvent A was 0.1% formic acid in water, solvent B was 0.1% formic acid in 80% acetonitrile): initial, 2% B; 0 min, 25% B; 80 min, 40% B: 90 min, 95% B; 95 min, 95% B; 100 min. The eluting peptides were analyzed in the data-dependent mode MSMS. A MS survey scan of 400–1600 *m*/*z* was performed in the Orbitrap at a resolution of 120,000 auto maximum injection time and the standard preset AGC target. The top speed mode was used to select ions for MS2 analysis with dynamic exclusion 20 s with a ± 10 ppm window. During the MS2 analyses, precursors were isolated using a width of 0.7 *m*/*z* and fragmented by HCD followed by Orbitrap analysis at a resolution of 50 000. HCD precursors were fragmented with a collision energy of 38% with auto maximum injection time and an AGC target of 250%.

Enriched Kac peptides were analyzed using Orbitrap Exploris 480 mass spectrometer (Thermo Fisher Scientific) coupled with an UltiMate 3000 RSLCnano system (Thermo Fisher Scientific). Peptides were loaded onto a tip column (75 μm diameter, 150 mm length) packed with reverse phase beads (3 μm/120 Å ReproSil-Pur C18 resin, Dr. Maisch HPLC GmbH). A 60-min gradient of 5 to 35% (v/v) from buffer A (0.1% (v/v) formic acid) to buffer B (0.1% (v/v) formic acid with 80% (v/v) acetonitrile) at a flow rate of 300 μL/min was used. The MS method consisted of a full MS scan from 350 to 1200 *m*/*z* at resolution 120,000, followed by data-dependent MS/MS scans with a first mass of 110 *m*/*z*, MS2 resolution of 45 000, isolation window of 0.7 *m*/*z*, and HCD collision energy of 36%. A dynamic exclusion duration was set to 60 s.

Enriched glycopeptides were analyzed using an Orbitrap Exploris 480 mass spectrometer coupled with an Ultimate 3000 UHPLC. Peptides were loaded *via* an Acclaim PepMap 100 trap column (C18, 5 μm, 100 Å; Thermo Scientific, San Jose, CA) onto a Waters BEH C18 column (100 μm × 100 mm) packed with reverse phase beads (1.7 μm, 120-Å pore size, Waters, Milford, MA). A 45-min gradient from 5 to 30% acetonitrile (v/v) containing 0.1% formic acid (v/v) was performed at an eluent flow rate of 500 nL/min. Data-dependent acquisition with a cycle time of 1s was used. The MS method consisted of a full ms1 scan (resolution: 60 000; AGC target: 300%; maximum IT: 50 ms; scan range: 350–1600 *m*/*z*) preceded by subsequent ms2 scans (resolution: 30 000; AGC target: 200%; maximum IT: 105 ms; isolation window: 1.3 *m*/*z*; first mass: 110m/z; NCE: 35%). To minimize repeated sequencing of the same peptides, the dynamic exclusion was set to 30 s and the “exclude isotopes” option was activated.

### Bioinformatic Data Processing and Statistical Data Analysis

Raw MS data for TMT-based proteomics were searched against a *Homo sapiens* (human) reviewed (Swiss-Prot) canonical protein database from UniProtKB (downloaded on 2023/11/09, with 20 428 protein entries) using MaxQuant (v2.4.10.0).^15,16^ Default parameters were used for the MaxQuant database search with reporter ion MS2 mode. The TMT reagent lot-specific reporter ion isotopic distributions were used as isotope correction factors for each set of TMT labeling data. The quantification data were then summarized and normalized using MSstatsTMT^17^ with the reference channels as bridge channel for different TMT experiments to generate quantitative protein group data matrix for further statistical analysis.

Lysine acetyl-proteomics data were processed using MaxQuant (v2.4.0.0)^15,16^ with the same UniProtKB human database (downloaded on 2023/11/09, with 20 428 protein entries). MaxQuant parameters were set the same as the proteomics data as described above, with the following exceptions: lysine acetylation (Acetyl (K)) was added as additional variable modification, and the maximum missing cleavage sites were set as 3. MSstatsPTM^18^ was then used to summarize and normalize the Kac site abundance data using the reference channels as bridge channel for different TMT experiments. To calculate the relative lysine acetylation levels on proteins, the proteomic data were used to adjust the Kac site abundance using MSstatsPTM as well. The proteome-adjusted abundance of all identified Kac sites in all groups was compared using a pairwise *t* test with FDR correction. The sites with FDR ≤ 0.05 and fold change (FC) ≥ 2 were deemed as significantly changed in this study.

Glycoproteomic data were processed by MSFragger (v3.7) implemented in Fragpipe 18.0 with an UniProtKB human database (downloaded on 2023/02/15, 20376 protein entries). Decoy and contaminants lists were added by using FragPipe. A default TMT10 workflow was used with changing TMT10 to TMT11 in the TMT interrogator tab. Two missed cleavages were allowed with +57.02146 on cysteine as fixed modification. + 15.9949 on methionine and 0.9840 on asparagine were set as variable. Mass tolerance was set to 20 ppm for both the precursor and fragment ions. Percolator was run with a 1% false discovery rate (FDR) filter and TMT-integrator filtered peptide spectral match and peptide with minimum probability of 0.9 for quantification. All identified glycopeptides with the N-X-S/T motif were then selected for further statistical data analysis.

Principal component analysis (PCA) was performed using all quantified proteins, Kac sites, or glycoproteins with no missing values.^19^ For partial least-squares–discriminant analysis (PLS-DA), all summarized and normalized data were uploaded to MetaboAnalyst^20^ for data filtering to keep variables with valid values in ≥50% samples and data imputation using k-nearest neighbors algorithm; the resulting data matrix was then used for PLS-DA to calculate the variable importance in projection (VIP) of each protein, Kac site, or glycoprotein to the model.^21^ VIP is a normalized value, and a VIP > 1 indicates a significant contribution to the PLS-DA model.^21^ A PLS-DA model with a goodness of prediction (Q2) > 0.4 was considered as acceptable in this study. Hierarchical clustering was performed using Perseus^22^ and R package *ComplexHeatmap*. Gene ontology enrichment analyses were performed using STRING with all identified proteins as the background for the calculation of false discovery rates (FDR).^23^

### Data Visualization

Experimental workflow was generated using BioRender (https://www.biorender.com/). Protein–protein interaction network was generated using STRING (https://string-db.org/). Boxplots, volcano plots, and functional enrichment dot plots were generated using R packages *ggplot2* and *ggpubr*. Heatmap, hierarchical clustering, and UpSet analysis and plotting were performed using the R package *ComplexHeatmap*. PCA and plotting were performed in R as well using R function *autoplot* and *prcomp*.

## Results

**Figure 1:**
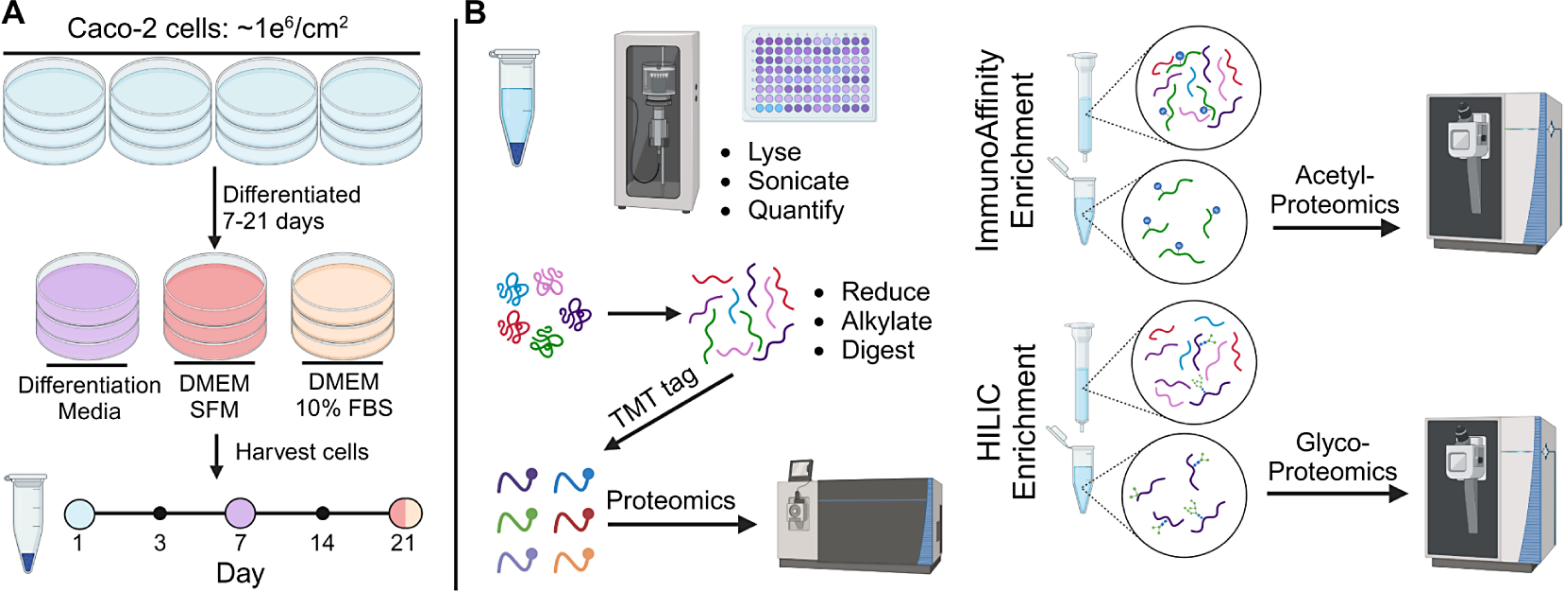
Graphic scheme depicting the cell culture and multiomics workflow of this study. Caco-2 cells were cultured in different media over 21 days with sample collection at different time points (A). Sample processing workflow used to prepare peptide samples for mass spectrometry (MS) analysis (B).

To characterize the Caco-2 cell differentiation, confluent cells were cultured using either regular Dulbecco’s Modified Eagle Medium (DMEM) growth media with serum (DFBS), without serum (SFM), or a commercial enterocyte differentiation media (DM, contains butyrate) for 21 days (Figure 1A). It is reported that DM can induce full cellular differentiation in 5–7 days.^8^ Accordingly, we found that at day 7, DM group cells formed dome structures, hallmark of Caco-2 cellular differentiation, but not yet for DFBS and SFM groups (SupplementaryFigure 1A–C). To examine and compare the molecular alterations at the proteome level, we collected samples throughout the course of differentiation at days 1, 3, 7, 14, and 21 (Figure 1A). The harvested cells were then lysed, digested, and labeled with isobaric TMT tags for multiplexed quantitative proteomic analyses. TMT-based protein quantitation is based on reporter ions at MS2 level and has been well demonstrated to be more accurate with less missing values when compared to label-free quantification (LFQ).^24^ Multiplexing of peptide samples also significantly reduced the needed amount of each sample and the cost of reagents for further enrichment of low-abundance modified peptides. In this study, a portion of the TMT-labeled peptide mixtures was fractionated with high pH reverse-phase fractionation and directly analyzed by LC-MSMS for total proteome measurement. The remaining peptide mixtures were further enriched by immunoaffinity approach for lysine acetyl-proteomics and hydrophilic interaction liquid interaction chromatography (HILIC) for glycoproteomics (Figure 1B).

### Quantitative Proteomics Demonstrates Differentiation of Caco-2 Cells

For multiplexed quantitative proteomics, a total of 54 samples (6 replicates for undifferentiated cells on day 1, and 4 replicates for all other groups) were randomized and assigned into six TMT11-plex experiments (Supplementary Table 1).^13^ To allow for cross-TMT experiment normalization, channels 1 and 11 of each TMT experiment were used to label a mixed reference sample. Each of the TMT experiment mixtures was then fractionated into 8 fractions and analyzed by mass spectrometry (MS). The resulting raw MS files were then processed with MaxQuant, which identified 45 555 peptides and 5038 protein groups. MSstatsTMT was then used for summarization and normalization, which resulted in the quantification of 4962 protein groups in total, and 3337 were quantified in all samples.

**Figure 2:**
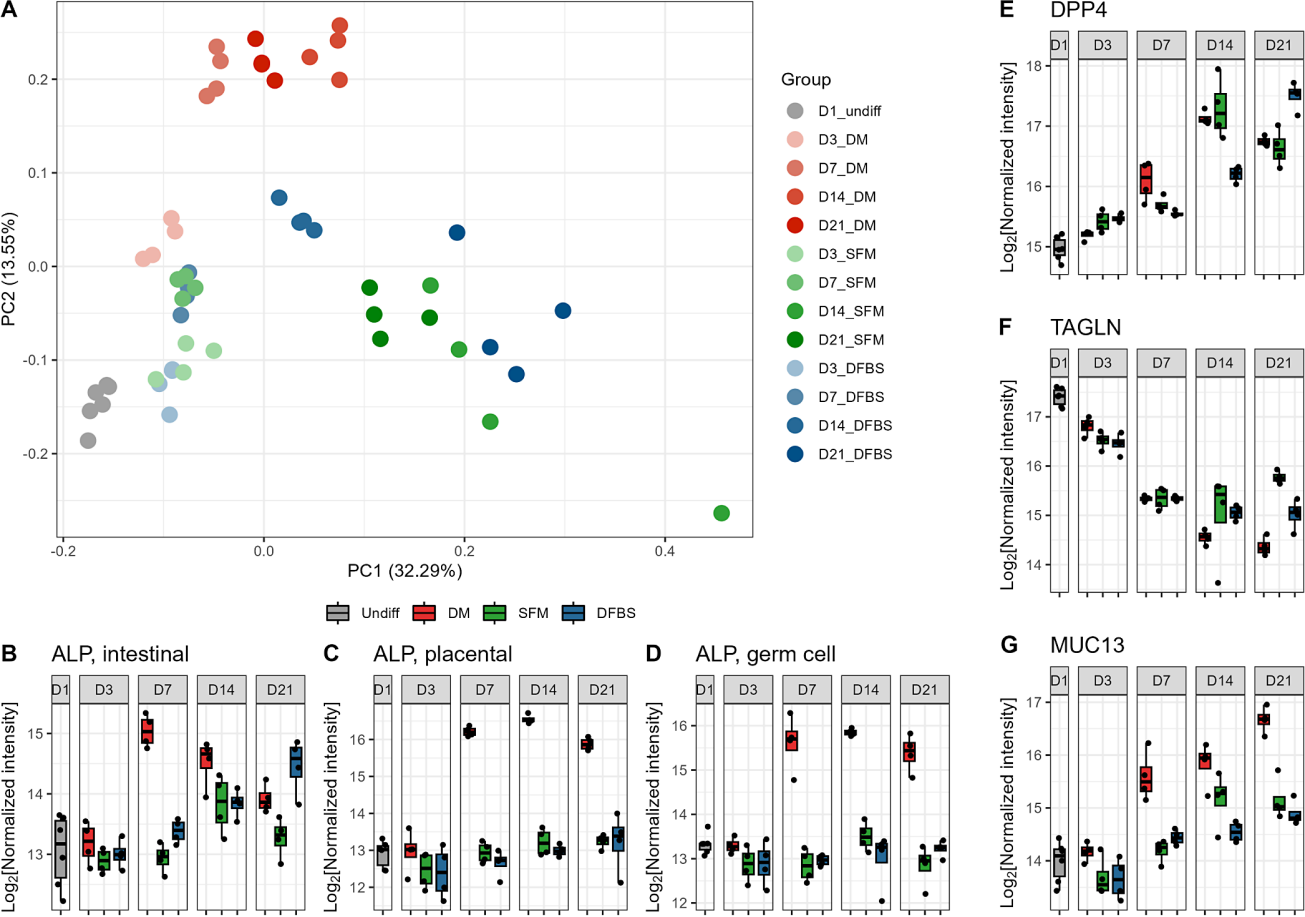
Proteomic overview and established markers of Caco-2 differentiation from carcinoma into intestinal epithelial-like cells. PCA score plot of quantified proteins across all samples (A). Log_2_(normalized intensity) of alkaline phosphatase (ALP) intestinal (B), placental (C), and germ cell (D) isoforms over time. Log_2_(normalized intensity) of other selected differentiation markers; DPP4 (E), TAGLN (F), MUC13 (G).

Principal component analysis (PCA) of the quantified protein groups in all samples was performed to examine the clustering patterns of undifferentiated Caco-2 cells compared with cells undergoing spontaneous and DM-induced differentiation over time (Figure 2A). The PCA score plot revealed distinct clustering of the different groups, indicating significant differences in their protein expression profiles. Undifferentiated and differentiated Caco-2 cells, as well as Caco-2 cells which underwent different differentiation protocols, over time clustered separately from each other. This observation suggests distinct proteomic changes in differentiated cells compared to undifferentiated cells, as well as distinct proteomic changes between spontaneous and DM-induced differentiated Caco-2 cells.

We then evaluated the expression patterns of known biomarkers for intestinal epithelium cell differentiation (Figure 2B-G). Alkaline phosphatase (ALP) activity has long served as a marker of Caco-2 cell differentiation.^25,26^ It is expected that protein expression levels would positively correlate with the alkaline phosphatase activity. In the DM group, a significant increase in intestinal type ALP abundance was observed as early as day 7 (adj. p-value 9.8 × 10^–13^) (Figure 2B). The DFBS group displayed a more gradual increase in ALP abundance over time, taking until day 21 to have a significant rise in ALP abundance (adj. p-value 5.8 × 10^–09^) (Figure 2B). Interestingly, we also noticed a significant increase in placental (adj. p-value 7.0 × 10^–17^) and germ cell type (adj. p-value 1.1 × 10^–11^) ALP abundance within the DM group that has been previously reported in studies exploring other butyrate-treated colonic adenocarcinoma cell lines (Figure 2C–D).^12,27^ This observation suggests a potentially broad effect of butyrate on the various ALP isoforms.

Additional established biomarkers for Caco-2 differentiation into intestinal epithelium-like cells observed in this study include an increase in dipeptidyl peptidase 4 (DPP4)^28,29^ (Figure 2E), a decrease in transgelin (TAGLN) (Figure 2F), an increase in mucin 13 (MUC13)^30^ (Figure 2G), and an increase in both thiosulfate sulfurtransferase (TST)^9^ and 3-hydroxybutyrate dehydrogenase 2 (BDH2)^9^ (Supplementary Table 2). Villin-1 (VIL1) and villin-2 (EZR) are both cytoskeletal proteins that are highly expressed in the brush border of intestinal epithelial cells, playing roles in maintaining the structural integrity and functionality of the microvilli, which increase the surface area for intestinal nutrient absorption and barrier function.^31,32^ We observed upregulation of villin proteins in the DM group at day 7, but not in DFBS and SFM groups which is worth further investigation (SupplementaryFigure 2A,B).

### Multivariate Statistics Identified Differentially Abundant Proteins in Differentiated Caco-2 Cells

**Figure 3:**
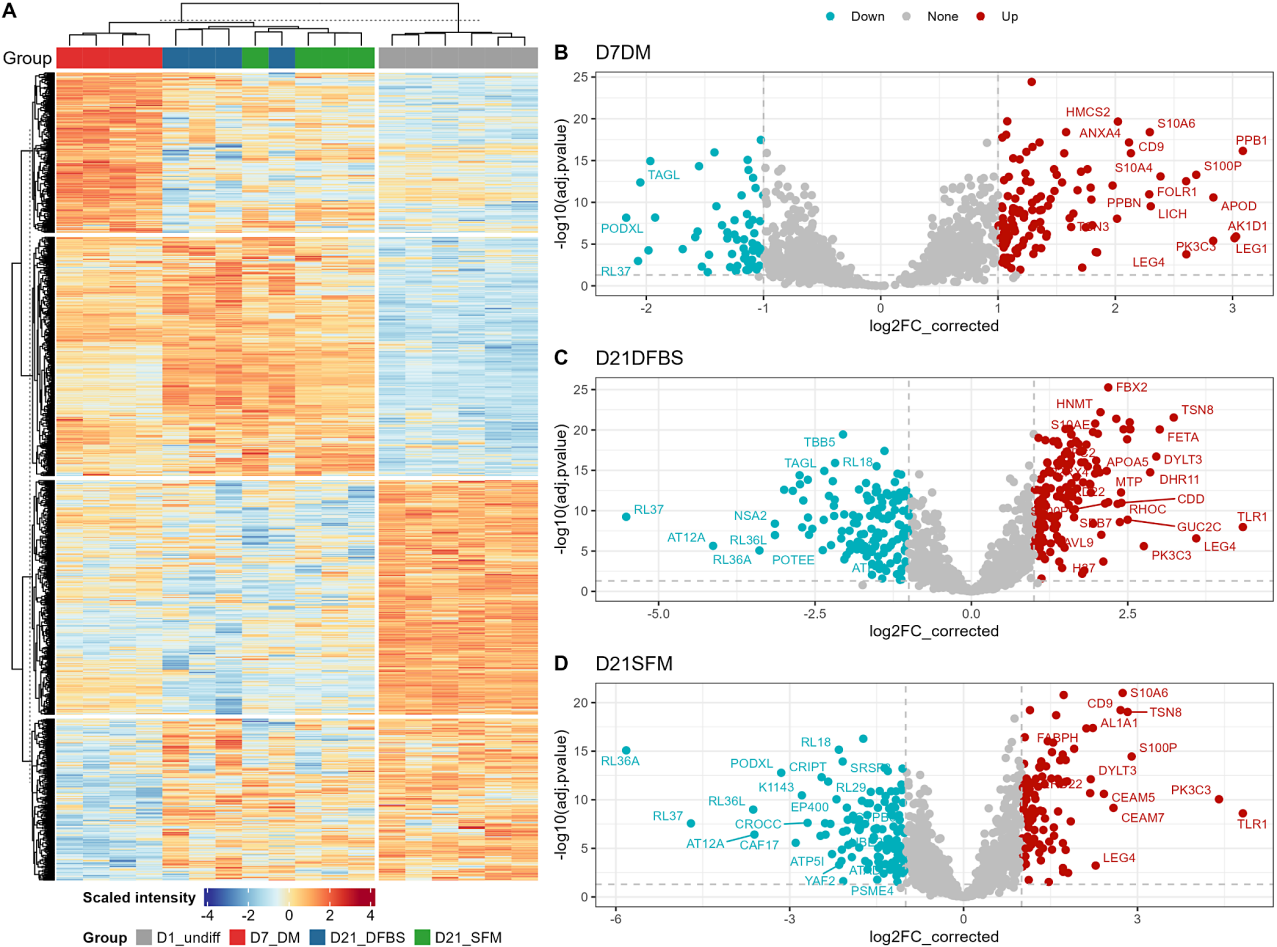
Differentially abundant protein expression of key Caco-2 cell differentiation groups. Heatmap and hierarchical clustering of samples using differentially abundant VIP ≥ 1 proteins (A). Volcano plots of Log2FC of all VIP ≥ 1 proteins, highlighting those with a fold change ≥2 and adjusted p-value ≤0.05, compare undifferentiated day 1 Caco-2 cells to cells grown in DM for 7 days (B), DFBS for 21 days (C), and SFM for 21 days (D).

To identify proteins that were altered during Caco-2 cell differentiation, we performed a partial least-squares-discriminant analysis (PLS-DA) for all quantified proteins in undifferentiated (D1_undiff) and differentiated cell groups (D7_DM, D21_DFBS, D21_SFM). Successful group discrimination model was established with a goodness of prediction (Q2) of 0.89 and an R2 of 0.99. By using a variable importance projection (VIP) cutoff of 1, a total of 1008 proteins were identified as differentially abundant proteins (Supplementary Table 2). Hierarchical clustering of the expression changes of all the VIP proteins indicated that 508 proteins were upregulated, and 500 proteins were downregulated in differentiated cells compared to undifferentiated samples (Figure 3A). Interestingly, 135 upregulated proteins in differentiated cells have a general trend of a higher extent of upregulation in DM than SFM and DFBS groups, despite less differentiation time used for DM (7 days compared to 21 days in the other two groups) (Figure 3A).

Among the upregulated proteins in differentiated Caco-2 cells when compared to undifferentiated cells and ranked highest in the PLSDA VIP analysis is phosphatidylinositol 3-kinase catalytic subunit type 3 (PIK3C3, VIP = 7.38), which was significantly changed in all groups (Figure 3A-C, SupplementaryFigure 2C and Supplementary Table 2). PK3C3 is recognized to be involved in multiple membrane-trafficking pathways, suggesting extensive membrane restructuring during Caco-2 differentiation. CD9 (VIP = 4.97), a cell surface glycoprotein and well-known exosome biomarker, was also among the highest VIP ranking significantly upregulated proteins and showed a steady increasing trend during the entire 21 days culturing in all three differentiation protocols (Supplementary Table 2 and Supplementary Figure 2D). We also observed that aldo-keto reductase family 1 member D1 (AK1D1, VIP = 5.99), galectin-1 (VIP = 6.24), and folate receptor alpha (FOLR1, VIP = 5.50) were among the proteins with highest VIP values but were only or more extensively upregulated in DM group (Supplementary Table 2 and SupplementaryFigure 2E–F).

**Figure 4:**
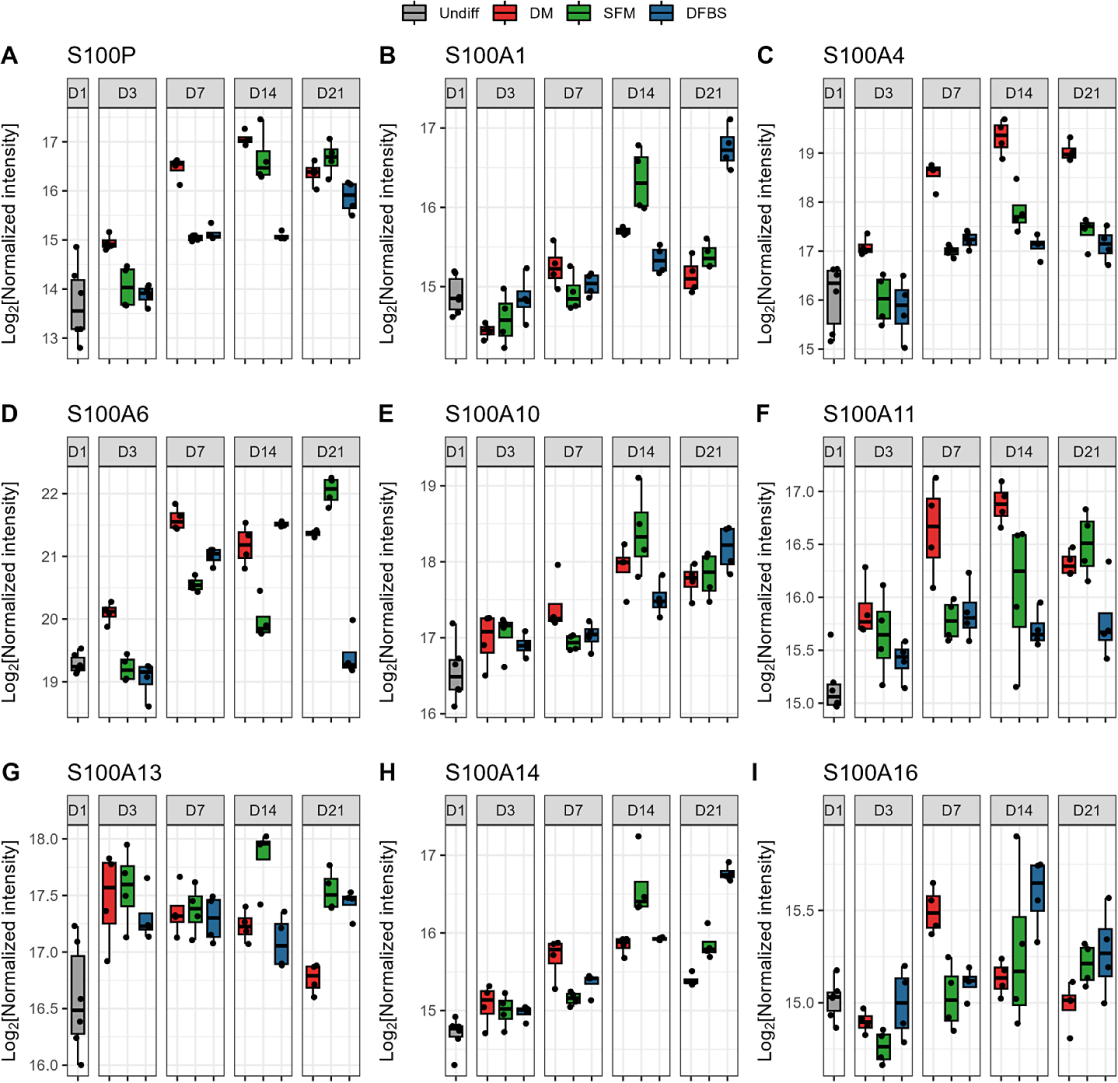
Differentially abundant protein expression of key Caco-2 cell differentiation groups. Log_2_(normalized intensity) of the following S100 protein family members over time: S100P (A), S100A1 (B), S100A4 (C), S100A6 (D), S100A10 (E), S100A11 (F), S100A13 (G), S100A14 (H), and S100A16 (I).

We identified four S100 family proteins with VIP values of more than 1, including S100A6 (VIP = 6.31), S100A4 (VIP = 5.25), S100P (VIP = 6.56), and S100A11 (VIP = 3.55). All of the quantified S100 family proteins showed upregulation with differentiation across both DM-induced and spontaneously differentiated groups (Figure 4A–I and Supplementary Table2). The expression levels of S100A4 and S100A11 significantly increased in DM group starting from day 7 when compared to other groups. The S100 protein family is a group of small, calcium-binding proteins that play diverse roles in cellular functions.^33^ In addition to S100 family proteins, annexins are another family of calcium-dependent proteins that are involved in diverse cellular processes, including membrane dynamics, vesicle trafficking, and signal transduction and can form protein complexes with S100 proteins in certain cellular contexts.^34^ Accordingly, we also found that many annexins, in particular annexin A6, were increased in DM-induced differentiated cells only, and the increase starts as early as day 3 of induction (SupplementaryFigure 2G–L).

In agreement with the retarded cell proliferation in differentiated cells, we found that the most significantly downregulated proteins include ubiquitin-conjugating enzyme E2 T (VIP = 4.74), large ribosomal subunit protein eL37 (RPL37, VIP = 5.28), eL42 (RPL36A, VIP = 4.80), and DNA-directed RNA polymerase I subunit RPA34 (CD3EAP, VIP = 2.69) (SupplementaryFigure 2M–P and Supplementary Table 2). We also found a significant decrease in the expression level of podocalyxin (PODXL, VIP = 5.14), an antiadhesive transmembrane glycoprotein, during Caco-2 cellular differentiation in all three protocols. Overexpression of PODXL has been reported to inhibit cell–cell interaction and be indicative of poor prognosis in colorectal cancer.^35^ The decrease in PODXL is therefore in agreement with the fact that differentiated Caco-2 cells have well established tight junctions.

### Functional and Pathway Alterations in Differentiated Caco-2 Cells

**Figure 5:**
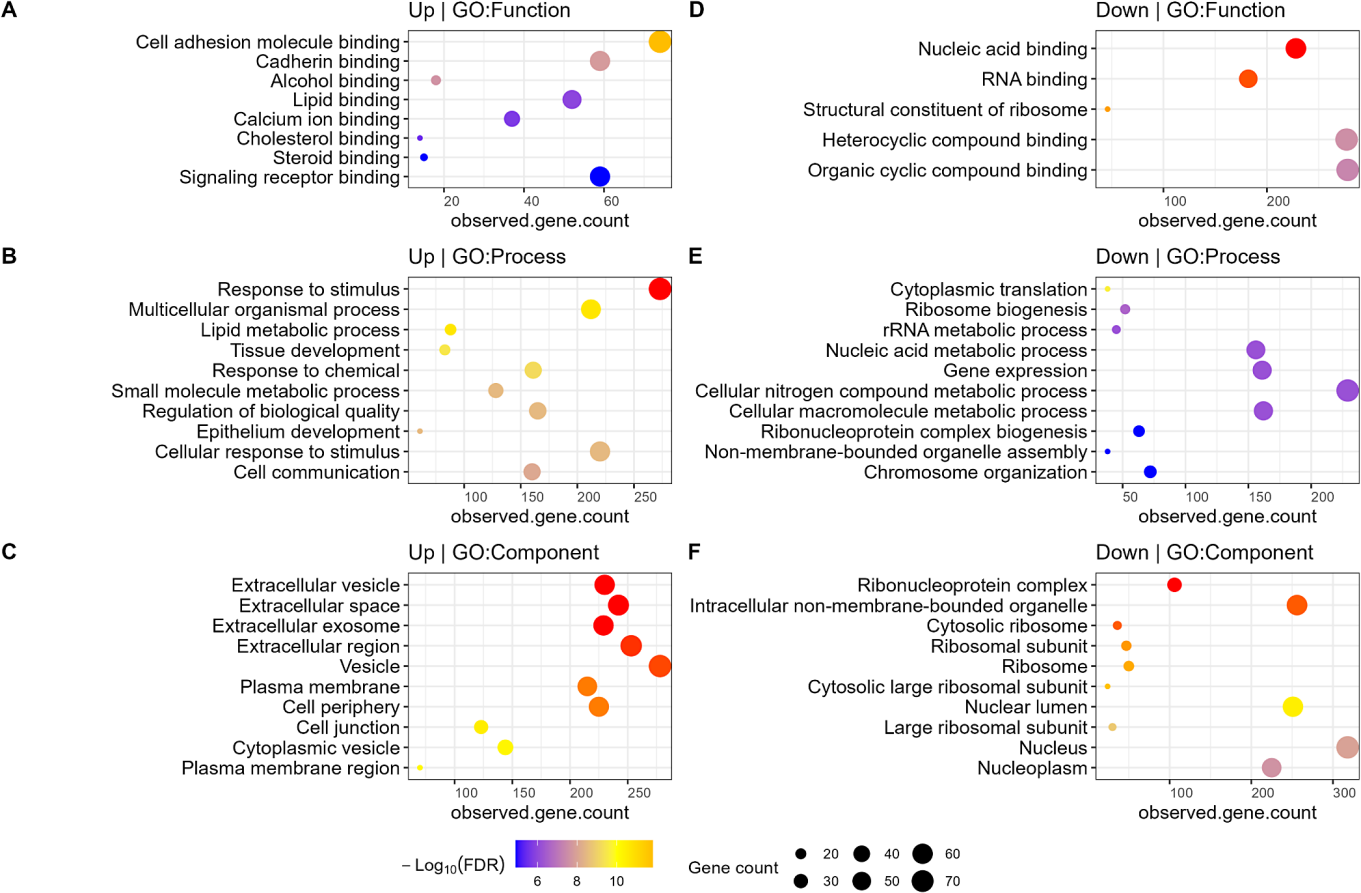
Clustering and functional enrichment analyses of differentially abundant proteins in differentiated cells compared to undifferentiated Caco-2 cells. Gene ontology enrichment analysis using STRING for PLSDA VIP ≥ 1 proteins that were upregulated (A–C) and downregulated (D–F), respectively, in differentiated groups (D7_DM, D21_DFBS, or D21_SFM).

To better explore the functional and pathway changes during differentiation, we then performed functional enrichment analysis for the up- or downregulated proteins in differentiated Caco-2 cells using the STRING database (Figure 5).

The upregulated proteins in the differentiated groups were enriched in functions related to cell adhesion molecule binding, cadherin binding, calcium ion binding, and other metabolite/small molecule/protein/lipid-binding activities (Figure 5A). Biological processes related to the responses to stimuli and chemicals, lipid metabolism, cell communication, and epithelial development were significantly enriched in the upregulated proteins in differentiated Caco-2 cells (Figure 5B). The upregulated proteins were also significantly enriched in cellular components, including extracellular vesicles, exosomes, plasma membranes, and cell junctions (Figure 5C). These observations indicate a shift in the cellular activity as it differentiates, including an increase in the formation of junctions and connections between cells, as well as the development of a more epithelial phenotype, which align with known cell changes postdifferentiation. A subset of the upregulated proteins in differentiated cells displayed a higher extent of upregulation within the DM group (Figure 3A). Functional enrichment analysis showed that these proteins were enriched in functions and pathways related to cell adhesion, epithelial differentiation, with a focus of proteins executing functions in extracellular vesicles, cell junctions, and around the cell membrane (SupplementaryFigure 3A–C).

The downregulated proteins in the differentiated groups were enriched in functions mainly involved in nucleotide binding, which would align with a decrease in cell replication (Figure 5D). In the differentiated groups, there was a decrease of processes related to translation, ribosome biogenesis, and nucleic acid metabolism (Figure 5E). From the cellular component enrichment analysis, the downregulated proteins were mainly enriched in the cytoplasmic space, nucleus, and intracellular organelles (Figure 5F). These pathway-level findings align with a progression away from cell division during differentiation. Overall, these functional- and pathway-level observations aligned with a phenotype change from replicating carcinoma cells to a more specialized, interconnected, epithelial cell phenotype.

### Glycoproteomic Alterations during Caco-2 Differentiation

Total proteome analysis demonstrated significant alterations of cell–cell interactions, which may involve extensive membrane protein changes. In order to deepen the analysis of these cell surface and highly glycosylated membrane proteins, we utilized HILIC to enrich glycopeptides prior to LC-MSMS analysis. Overall, 1070 N-glycosylated sites were identified in this study, 731 of which were quantified in ≥50% of the samples. The PCA score plot of the expression levels of quantified N-glycosylated protein sites matched clustering patterns seen in the proteomics analysis, showing over time distinct clustering of the different groups (Supplementary Figure 4A). This suggests that both spontaneous and DM-induced differentiation induced changes of N-glycoprotein expression at the cell surface compared with undifferentiated cells. The findings in this study are also in agreement and supplement the observations in a recent integrated glycomic and proteomic study, which demonstrated significant glycan structural alterations in butyrate-stimulated Caco-2 cell differentiations.^12^

**Figure 6:**
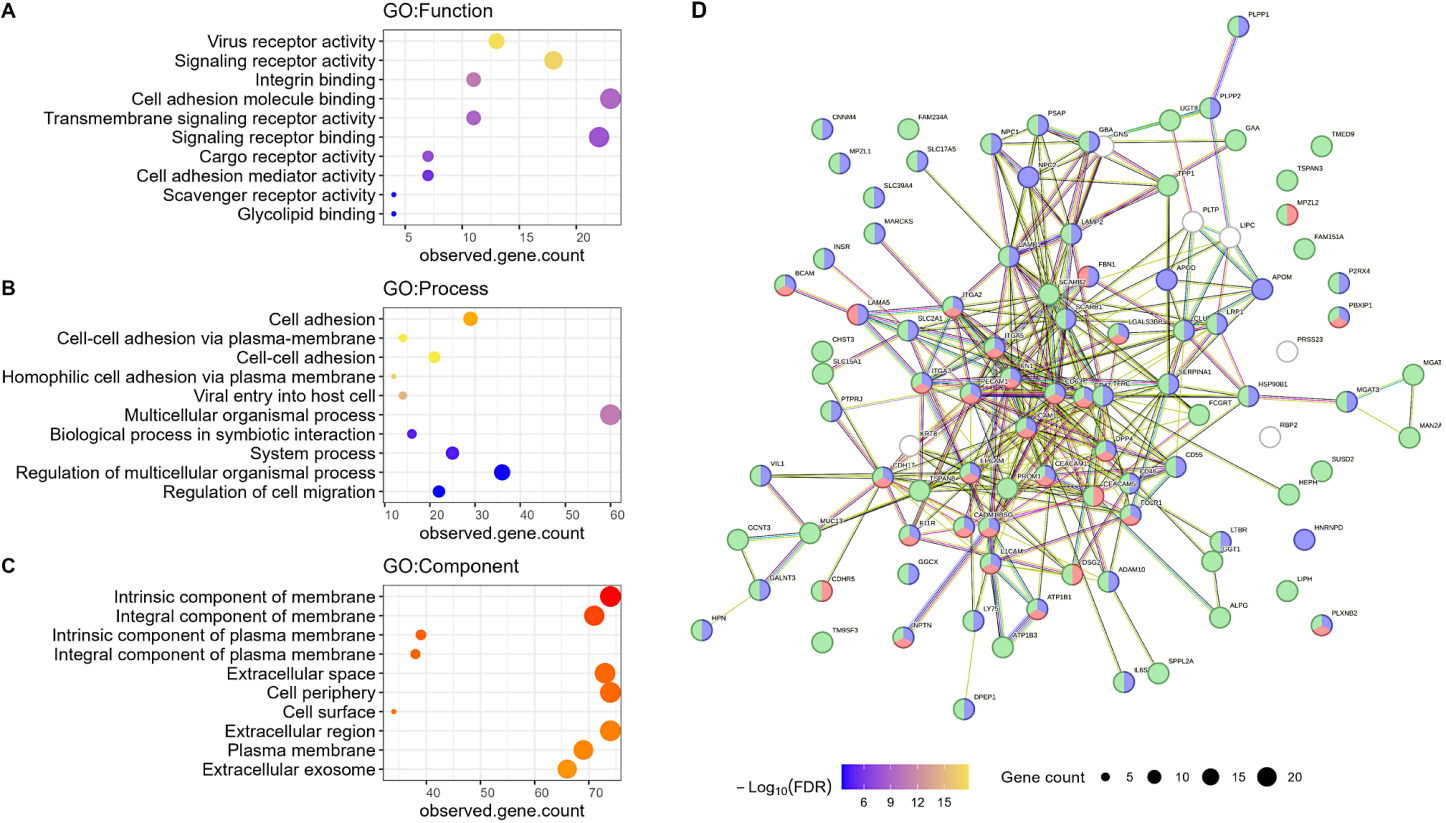
Functional enrichment and protein–protein interactions of differentially abundant N-glycosylated proteins in differentiated cells. Gene ontology enrichment for biological functions (A), processes (B), and cellular components (C) were performed using STRING with PLSDA VIP ≥ 1 proteins upregulated in differentiated Caco-2 cells. Protein–protein interaction maps were generated using STRING (D) highlighted enrichment terms are cell adhesion (GO: 0007155, Red), response to stimulus (GO:0050896, Blue), and membrane (GO: 0016020).

A PLS-DA for key groups using quantified *N*-glycosylation site data showed similar clustering results to that of the proteomics data set. Of 165 differentially abundant *N*-glycosylated sites identified with a VIP threshold over 1, 159 of them were upregulated in differentiated Caco-2 cells (Supplementary Table 3). These differentially abundant sites were then subjected to functional enrichment analysis using the STRING database (Figure 6). As expected, the most significant enriched cellular components are related to membranes and extracellular spaces (Figure 6). Significant molecular function enrichment was also obtained for receptor activity and cell adhesion molecule binding, in particular, virus receptor and signaling receptor activities (Figure 6A). Interestingly, in addition to the cell adhesion processes, biological processes involved in symbiotic interaction and viral entry into the host cell were among the most significantly enriched processes (Figure 6B), indicating cellular adaptation for host–microbiome interactions. Similar to the differential abundant proteins at the proteome level, the extracellular space and exosome are also among the most significantly enriched cellular components (Figure 6C). STRING analysis also demonstrated extensive protein–protein interactions among the differentially abundant *N*-glycosylated proteins (Figure 6D), suggesting close crosstalk between the pathways associated with Caco-2 differentiations. We identified several proteins with multiple sites that were upregulated in differentiated cells, such as lysosomal associated membrane protein 1 (LAMP-1, 10 sites), carcinoembryonic antigen-related cell adhesion molecule 1 (CEACAM-1, 7 sites), prosaposin (SAP, 4 sites), DPP4 (4 sites), CEACAM-5 (7 sites), and galectin 3 binding protein (LG3BP, 6 sites) (Supplementary Table 3). These findings in particular the pathway changes further demonstrated an increase and the involvement of glycosylation in regulating cell adhesion, signaling pathway, and membrane transport upon transition of Caco-2 to an intestinal-epithelial-like phenotype, providing further insights into the application of differentiated Caco-2 cells to study host–microbiome interactions.

### Lysine Acetylation Was Involved in Caco-2 Cell Differentiations

Protein acetylation plays important roles in regulating translation, transcription, metabolism, and immunity in both eukaryotes and bacteria. Aberrant protein acetylation levels have been implicated in various cancers, such as colorectal cancer,^36,37^ and dysbiotic host–microbiome interactions in diseases, such as Crohn’s disease.^38^ To this end, we performed lysine acetyl-proteomics analysis for Caco-2 differentiation. Following identification and quantification with MaxQuant, MSstatsPTM was used to summarize the acetyl-proteomic data and to perform abundance adjustment using the total proteome data. In this study, multiple lysine acetylation (Kac) modifications on one identified modified peptide sequence were considered as an individual site (highly acetylated sites; mainly present on histone proteins). Altogether, this study identified 5994 Kac sites on 2136 proteins, with the most abundant sites on histone protein H4, H31, H2B type 1-K, as well as acyl-CoA-binding protein, chloride intracellular channel protein 1 (CLIC1), malate dehydrogenase, adenosylhomocysterinase, and annexin A4 (Supplementary Table 4). A total of 3459 Kac sites were quantified in >50% samples and PCA showed that the undifferentiated Caco-2 cells clustered together while the cells undergoing differentiation shift away from undifferentiated cells by time regardless of differentiation protocols, suggesting altered-lysine acetylproteomes of differentiated Caco-2 cells (SupplementaryFigure 5A).

**Figure 7:**
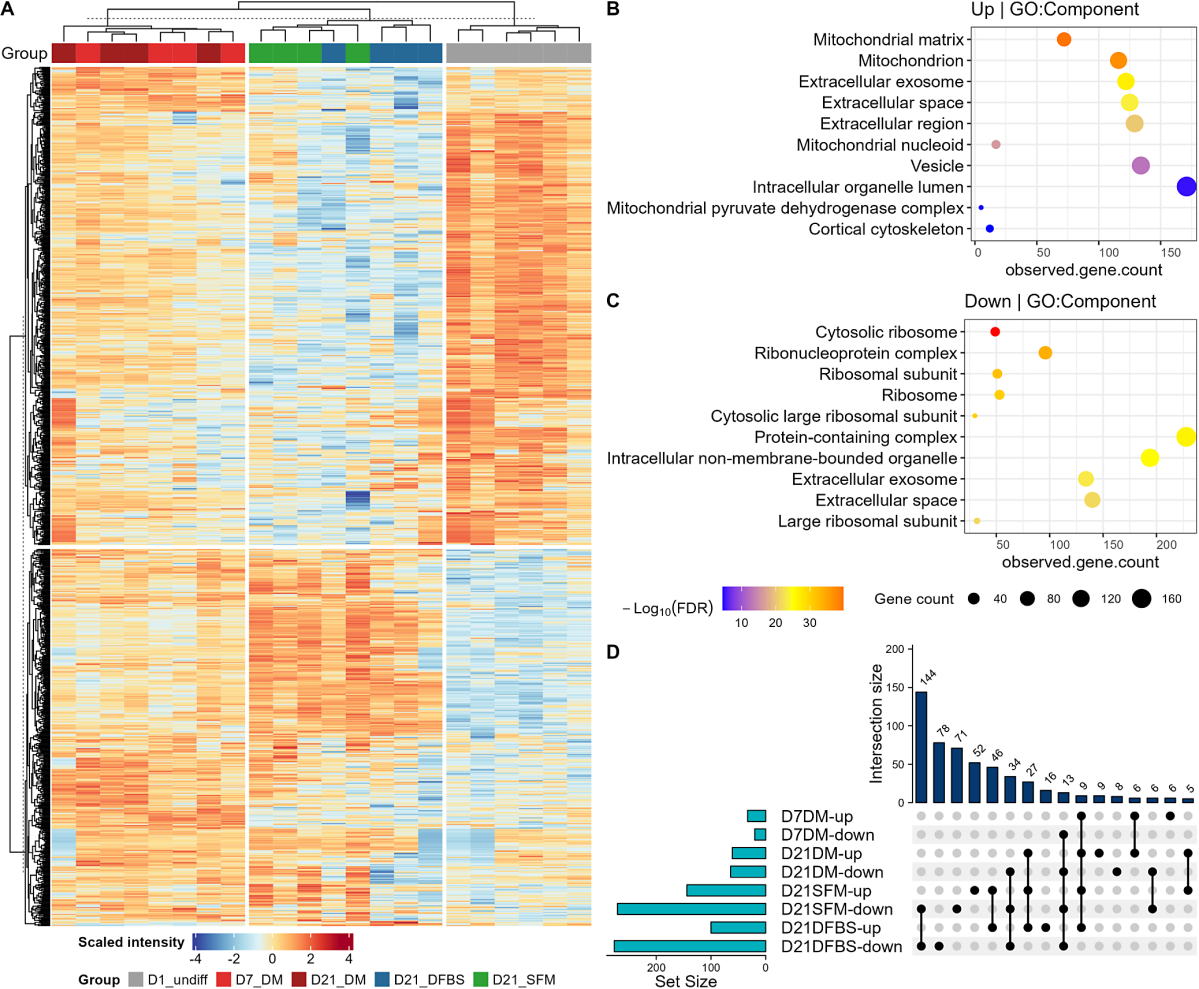
Overall trends in acetylated proteins of key differentiated Caco-2 cell groups compared with undifferentiated cells. Heatmap and clustering of PLSDA VIP ≥ 1 lysine-acetylated proteins (A). Clustering and functional enrichment analysis of differentially abundant proteins in differentiated cells compared to undifferentiated Caco-2 cells. Gene ontology enrichment analysis using STRING for PLSDA VIP ≥ 1 of components that were upregulated (B) and downregulated (C) in differentiated groups (D7_DM, D21_DFBS, or D21_SFM). Upset plot indicating overlap of PLSDA VIP ≥ 1, FC ≥ 2, adjusted p-value ≤0.05, unique lysine-acetylated protein/protein sites between selected differentiated groups compared to undifferentiated Caco-2 cells (D).

To identify differentially abundant Kac sites, we used PLS-DA for the analysis of five representative groups, including D7_DM, D21_DM, D21_SFM, and D21_DFBS, which successfully (Q2 = 0.59, R2 = 0.98) identified 914 differentially abundant Kac sites with a VIP threshold of 1 (Figure 7A and Supplementary Table 4). Among the 914 Kac sites, 449 sites were upregulated, and 465 were downregulated in differentiated Caco-2 cells compared to undifferentiated cancerous cells (Figure 7A). Pathway enrichment analysis showed that the proteins with upregulated Kac sites were mainly involved in mitochondria and catabolic metabolism processes, while the proteins with downregulated Kac sites were enriched in functions related to ribosome, translation, biosynthesis, and nucleic acid binding (Figure 7B–C, SupplementaryFigure 6A–D).

Among the 914 differentially abundant Kac sites, 878 have corresponding total protein abundance quantified in proteomic data set (Supplementary Table 4). Following adjustment using the total protein abundance, 551 Kac sites were deemed as having significantly altered acetylation levels with a threshold of fold change (FC) ≥ 2 and FDR-adjust *p* value ≤0.05. Among the 551 differentially abundant Kac sites, 53 sites (20 down, 33 up) were significantly changed in D7_DM, 125 sites (64 down, 61 up) in D21_DM, 415 sites (271 down, 144 up) in D21_SFM, and 377 sites (277 down, 100 up) in D21_DFBS groups compared to undifferentiated Caco-2 cells (Figure 7D). The majority of the Kac sites showed consistent changing direction across all four comparisons when overlapped; only 8 sites showed opposite directions (Figure 7D). In agreement with the sample clustering in the PCA score plot, the largest overlap was found to be between D21_DFBS and D21_SFM (144 down, 46 up) (Figure 7D). Sixty-one sites (34 down, 27 up) were found to be consistently regulated in the D21_DM, D21_DFBS, and D21_SFM groups (Figure 7D). There were 9 Kac sites being upregulated and 13 downregulated in all four groups, including D7_DM (Figure 7D, Supplementary Table 4).

Histones are among the most well-known abundant and highly acetylated proteins in cells. We identified 13 differentially abundant Kac sites on 6 histone proteins (H1, H1a, H2A, H3.2, H3.3, and H4) that were significantly altered (FC ≥ 2, adj. p-value <0.05) in at least one comparison (Supplementary Table 4). The acetylation levels were decreased for most of the histone acetylation sites, except for two (K6/9 and K9/13/17) on H4 that were upregulated in the D7_DM group. The Kac site K28/37 on histone H3.2 was significantly downregulated in all differentiation groups, except D7_DM. The acetylation level of K6/10 of histone H2A was the most significantly decreased in differentiated cells in both D21_SFM (log2FC = −4.38) and D21_DM (log2FC = −4.51) groups (Supplementary Table 4). Histone acetyltransferase p300 (p300 HAT) was also a highly acetylated protein with one triple-Kac peptide (K1542/1546/1549) identified and significantly upregulated in all differentiation groups with a log2FC > 1.55 and VIP value of 3.85 (Supplementary Table 4). HAT p300 has been previously reported to be a transcription factor coactivator and play important roles in maintaining intestinal homeostasis, cellular differentiation and prevent tumorigenesis in the intestine.^39^ The findings in this study suggest that lysine acetylation itself might be involved in regulating the function of p300, contributing to the maintenance of intestinal cell proliferation/differentiation.

In addition to p300, the Kac sites on heat shock protein HSP90 (K255), actin-related protein 2 (ARP2, K299), and annexin A5 (K79) were identified as upregulated in all differentiation groups (Supplementary Table 4). We found three Kac sites (K9, K201, and K246) on the aldo-keto reductase family 1 member C1 protein, which were upregulated in particular in DM groups (both day 7 and day 21). On the contrary, Kac sites on ATP synthase (various on different subunits), chloride intracellular channel protein (CLIC1, K135), and annexins A2 (K47) were more prominently upregulated in D21_DFBS and D21_SFM groups. Some of the most downregulated Kac sites in all differentiation groups were on hepatoma-derived growth factor (HDGF, K105), alpha-enolase (K406, K335), glutathione S-transferase omega-1 (K101), vasodilator-stimulated phosphoprotein (VASP, K71), and CD9 antigen (K179) (Supplementary Table 4).

## Discussions

Differentiated monolayer Caco-2 cells are commonly used as an *in vitro* model for studies exploring the intestinal absorption and reactions to various drugs or compounds. Accordingly, prior studies have explored some of the molecular changes between undifferentiated and differentiated Caco-2 cells through transcriptomics and proteomics, with most focusing in spontaneous differentiation.^6,9,12^ Microbial butyrate is known to induce rapid Caco-2 cell differentiation. Accordingly, a recent proteomic and *O*-glycomic study has compared spontaneous or butyrate-induced Caco-2 differentiation and demonstrated that butyrate-stimulated differentiation was correlated with higher expression of sialylated *O*-glycan and lower fucosylation.^12^ Differentiated Caco-2 cells have also been applied to study intestinal epithelial interactions with biotherapeutics, microbiota byproducts (*e.g.*, short-chain fatty acids), or microbiota themselves more recently. Due to the fact that Caco-2 cells are easy to culture, it holds potential for use as a microbiome-based therapeutic assessment tool, attracting interest in characterizing and understanding the molecular alterations during its differentiation over time with a focus on host–microbiome interactions.^3^ In addition to the involvement in cancer development and anticancer therapy, lysine acetylation has been demonstrated to be implicated in host–microbiome interactions in diseases, such as Crohn’s disease.^38^ However, to the best of our knowledge, there are no lysine acetylproteomic investigations when comparing spontaneous and DM-induced Caco-2 differentiations. Therefore, to better understand the Caco-2 cell differentiation processes, this study applied proteomic, lysine acetyl-proteomic, and glycoproteomic approaches to directly compare three commonly used Caco-2 differentiation protocols, including serum-containing and serum-free growth medium-induced spontaneous differentiation and butyrate-containing DM medium-induced rapid differentiation.

We showed that both spontaneous and DM-induced differentiated Caco-2 cells displayed overall proteomic, lysine acetyl-proteomic, and glycoproteomic patterns moving away from those of undifferentiated cells. Over the course of the time series, the proteomic clustering patterns of the DM group exhibited notable differences with a more stable clustering pattern after 7 days, indicating that the differentiation process was either complete or minimally changing by 7 days, ultimately reaching a steady-state of the cells. This aligns with the timeline of corresponding published butyrate-induced differentiation methods.^8,12^ In contrast, both spontaneous differentiation groups (DFBS and SFM) continued to cluster further apart from the undifferentiated cells with increasing time up until the last 21-day time-point tested.^6,9,12^ These results suggest ongoing proteomic and post-translational changes associated with the progression of spontaneous differentiation take longer to reach their respective plateaus or a more steady-state cell phenotype. This was also seen visually as by day 7, the DM group showed extensive dome formation, whereas the DFBS and SFM groups were not as extensive at the same early time point. We also found that although there are some differentially abundant proteins between DFBS and SFM groups at day 21, the presence of FBS in spontaneously differentiated groups did not alter biological processes or functions that are key to epithelial differentiation compared to the serum-free group (Supplementary Table 5, SupplementaryFigure 7).

We also demonstrated through pathway analysis an increase in stimuli response proteins/modified proteins, which are represented at the cell surface, in both cellular and extracellular membranes (vesicles, exosomes). This is reflected by increases in proteins with roles in membrane-trafficking pathways and extracellular vesicles, such as PIK3C3 and CD9. This reinforces that there are changes within the cell membrane as expected alongside the major morphological changes from Caco-2’s primary cancerous phenotype into an epithelial-like cell. Although a change in secreted vesicle quantity and size was not observed in preliminary investigations (data not shown), enriched pathways such as stimuli response, molecule binding, cell communication, and receptor activity suggest an increase in cell-to-cell cross-talk, as well as increased interactions with the environment, such as chemicals and microbes, as the cells differentiate. This likely ties into the vital symbiotic relationship between human intestinal processes and the gut microbiome which reside within it.^40^ Glycoproteomics data also highlighted an increase in biological process involved in symbiotic interaction, and our study observed a significant increase in proteins and/or their altered PTM levels that are known to interact with and play key roles in host–microbiome homeostasis such as multiple galectins (Gal-1, Gal-2, Gal-3, Gal-4, and Gal-7) and folate-receptor alpha subunit (Supplementary Table 2–4).^41^

An advantage of quantitative proteomics is to quantify multiple proteins, such as biomarkers, in a nontargeted manner. Accordingly, this study was able to quantify most known biomarkers of Caco-2 differentiation into intestinal epithelium-like cells, including ALP-intestinal type, MUC13, DPP4, TST, BHD2, and TAGLN, which all followed expected trends.^9,28 −30^ Intestinal-type ALP is the most well-known biomarker for intestinal cellular differentiation.^25,26^ We showed that the protein abundance of intestinal-type ALP gradually increased along with differentiation, with the DM group peaking at day 7, while SFM and DFBS groups peaking at days 14 and 21, respectively (Figure 2B). This trend was in agreement with the overall proteome pattern as well as the microscopic examination, indicating the rapid and efficient differentiation of Caco-2 cells using DM medium. Interestingly, in addition to the intestinal type ALP, we as well as a previous study both found that the two other types of ALPs (*i.e*., placental type and germ cell type) were significantly upregulated upon DM-induced differentiation at as early as day 7, but not in spontaneous differentiations (Figure 2C–D).^27^ These three types of ALPs may all contribute to the increased alkaline phosphatase activity in differentiated Caco-2 cells as generally measured using a *p*-nitrophenyl phosphate (pNPP)-based colorimetric assay. In addition to ALPs, DM-induced differentiation displayed higher abundance of MUC-13 at day 7 as well as at days 14 and 21. Similarly, villin proteins, the key components of intestinal villi structure, were found to be significantly upregulated in the DM group at day 7, but not in DFBS and SFM groups at day 7 or 21.^31,32^ Altogether, these findings suggest that full and efficient Caco-2 differentiation into epithelial-like intestinal cells can be rapidly (within 7 days) achieved with butyrate-containing commercially available DM medium.

Protein acetylation plays important roles in regulating translation, transcription, metabolism, and immunity in both eukaryotes and bacteria and has been implicated in multiple cancers, making it a molecular mechanism of interest in characterizing the Caco-2-derived intestinal model.^38^ Unlike proteomics, which showed more uniform changes across differently differentiated groups, it was found that there was only 53 and 125 significantly changed acetylation sites in the DM group at days 7 and 21, respectively, which is quite low compared to the 415 differentially abundant sites in the SFM group and 377 sites in the DFBS group at day 21. This mild change of Kac sites in DM group may be due to the presence of butyrate in the DM medium, as butyrate is a known histone deacetylase (HDAC) inhibitor.^42^ We found that, along with the Caco-2 cell differentiation across groups, most Kac sites on histone proteins (the most abundant Kac proteins) were downregulated, indicating a decreasing trend of overall lysine acetylation level in differentiated cells compared to cancerous cells. Accordingly, more downregulated Kac sites than upregulated Kac sites were observed in spontaneous differentiation groups (SFM and DFBS) (Figure 7A). The reduced histone protein Kac levels in differentiated Caco-2 cells, which favor the closed chromatin conformation, coincide with the proteomic observations showing downregulated translation and proliferation related protein expressions.

The HDAC inhibitor butyrate in DM medium reversed this trend of overall Kac levels by increasing some Kac proteins, in particular, at day 7 when more upregulated Kac sites than downregulated were obtained. These overall Kac protein changes were also reflected in the PCA score plot where the D7_DM and D21_DM groups clustered between the undifferentiated and spontaneous differentiation groups (D21_SFM and D21_DFBS). Of the Kac sites that are upregulated in differentiated Caco-2 cells identified in this study, they were found to be enriched in mitochondria, stress response-related proteins (such as HSP90 and ARP2), and ion transport or binding functions (such as CLIC1, annexins, and S100 family proteins). These findings indicate that protein acetylation may be involved in regulating intracellular homeostasis as well as cell–cell interactions during differentiation. It is known that calcium (Ca^2+^) plays an important role in maintaining intestinal homeostasis, including signaling and transport which are key processes in nutrient sensing, transport processes, and endocrine regulation.^43−48^ Proteomic data in this study also showed an upregulation of calcium ion binding functions as well as cadherin binding functions (which require calcium ions to function in cell adhesion/junction formation), reinforcing the importance of calcium within intestinal-like cells. The S100 protein family of Ca^2+^ binding EF-hand proteins play roles in both intracellular and extracellular regulation.^33,49^ These include functions in differentiation, Ca^2+^ homeostasis, metabolism, and immune response.^49^ This study demonstrated an association between Caco-2 differentiation and the upregulated expression of multiple S100 family proteins. This suggests a change in calcium signaling within Caco-2 cells during differentiation, with S100 proteins playing a role in the altered signaling pathways. Additionally, some annexins which are a family of non-EF-hand Ca^2+^ binding proteins, showed a similar association with Caco-2 differentiation. Annexins can form complexes and interact with S100 protein family proteins to play roles in membrane and vesicle trafficking and organization.^34,50^ We observed enrichment of some annexins, changes in multiple Kac sites on several annexin proteins (A2, S4, A5, and A11), as well as a functional enrichment of membrane and vesicle/exosome components for both upregulated proteins and Kac sites. Accordingly, cancerous cells (including colorectal cancer cells) display altered calcium signaling, which has become a target in cancer therapeutics, as calcium signaling regulates cell division, motility, and death with key roles in cancer cell migration and invasion.^51,52^ While the relationships may be complicated, the findings in this study may indicate a shift in calcium signaling during Caco-2 differentiation, and protein lysine acetylation may regulate this molecular process and influence the dynamic cellular changes occurring during Caco-2 cell differentiation.

In summary, this study offers a comprehensive characterization for Caco-2 cell differentiation using proteomics, acetyl-proteomics, and glycoproteomics. We confirmed the expected protein biomarker trends in both DM-induced and spontaneously differentiated Caco-2 cells. We also demonstrated that, regardless of differentiation methods, the differentiated Caco-2 cells are featured with a decrease of proteins related to translation and an increase of proteins with functions in cell adhesion, response to stimuli, cell signaling, epithelium development, as well as the extracellular vesicles/space. This study also provided crucial insights into the roles of lysine acetylation in Caco-2 differentiation and demonstrated an upregulation of lysine-acetylated proteins related to mitochondria functions. These findings reflect a shift of differentiated Caco-2 cells toward a more interconnected epithelial-like cell network and the development of cellular functionalities commonly associated with host–microbiome interactions. Compared to spontaneous differentiation, full and efficient differentiation can be achieved within 7 days using butyrate-containing differentiation medium for culturing the cells. We acknowledge that this is an aerobic culture model and more intricate microfluidic systems would be required for coculture of Caco-2 cells with gut microbiota, which contain many strict anaerobes. However, future applications of this model may include the assessment of microbiome-derived products and the identification of actionable molecular biomarkers that are indicative of the benefits and/or risks of various microbiome products on the host. Altogether, this study improved our understanding of the Caco-2 differentiation processes, which will aid its application in biomarker and drug target discovery, the study of host–microbiome interaction, and assay development for quality assessment of GI tract-directed therapeutics moving forward.

## Data Availability

All MS proteomics data that support the findings of this study have been deposited to the ProteomeXchange Consortium *via* the PRIDE partner repository with the data set identifier PDX049082, PDX049087, and PDX049091.

## Supporting Information Available

Outline of sample distribution across TMT experiments and labels used (Table S1); microscopic images comparing cell morphology during differentiation (Figure S1); differential protein expression of selected proteins in key Caco-2 cell differentiation groups (Figure S2); functional enrichment analysis of proteins upregulated in the D7DM group as compared to D21DFBS and D21SFM groups (Figure S3); overall trends in N-glycosylated proteins of Caco-2 differentiation (Figure S4); overall trends in acetylated proteins of key differentiated Caco-2 cell groups (Figure S5); functional enrichment of lysine-acetylated proteins in differentiated cells (Figure S6); differences between spontaneously differentiated Caco-2 cells cultured using either DFBS or SFM media at day 21 (Figure S7)(PDF)
Compilation of proteomic data (Table S2)(XLSX)
Compilation of glycoproteomic data (Table S3)(XLSX)
Compilation of lysine acetyl-proteomic data (Table S4)(XLSX)
Compilation of day 21 proteomic data for spontaneously differentiated groups (Table S5)(XLSX)
