# Supplementary material for: Multilevel Proteomic Profiling of Colorectal Adenocarcinoma Caco-2 Cell Differentiation to Characterize an Intestinal Epithelial Model
Source: J Proteome Res. 2024 May 29;23(7):2561–75. doi: 10.1021/acs.jproteome.4c00276 (PMC11232098; doi:10.1021/acs.jproteome.4c00276)
Supplement: Supplementary file 1 — pr4c00276_si_001.pdf [file pr4c00276_si_001.pdf]

Multilevel proteomic profiling of colorectal adenocarcinoma Caco-2 cell differentiation to characterize an intestinal epithelial model

Emily EF Fekete[1], Angela Wang[1], Marybeth Creskey[1], Sarah E Cummings[1], Jessie R Lavoie[1], Zhibin Ning[2,3], Jianjun Li[4], Daniel Figeys[2,3], Rui Chen[4], Xu Zhang[1,2]**

[1]Regulatory Research Division, Biologic and Radiopharmaceutical Drugs Directorate, Health Products and Food Branch, Health Canada, Ottawa K1A 0K9, Canada; [2]Department of Biochemistry, Microbiology and Immunology, Faculty of Medicine, University of Ottawa, Ottawa K1H8M5, Canada; [3]School of Pharmaceutical Sciences, Faculty of Medicine, University of Ottawa, Ottawa K1H8M5, Canada; [4]Human Health Therapeutics Research Centre, National Research Council Canada, Ottawa K1A0R6, Ontario, Canada.

Correspondance: X.Z., xu.zhang@hc-sc.gc.ca; R.C., rui.chen@nrc-cnrc.gc.ca

Supplementary information:

Supplementary Table S1.

Supplemental Figure 1

Supplemental Figure 2

Supplemental Figure 3

Supplemental Figure 4

Supplemental Figure 5

Supplemental Figure 6

Supplemental Figure 7

Supplemental Table 1. Outline of sample distribution across TMT experiments and labels used.

**Channels**	**Mixture 1**	**Mixture 2**	**Mixture 3**	**Mixture 4**	**Mixture 5**	**Mixture 6**
TMT^{10}-126	Pool	Pool	Pool	Pool	Pool	Pool
TMT^{10}-127N	D7-SFM-1	D3-DM-1	D3-DFBS-1	D21-SFM-4	D14-SFM-2	D14-DM-4
TMT^{10}-127C	D1-undiff-3	D7-DFBS-1	D7-SFM-3	D14-DFBS-2	D7-SFM-4	D3-SFM-4
TMT^{10}-128N	D14-DFBS-1	D1-undiff-2	D14-DM-1	D3-SFM-2	D3-DM-3	D7-DM-1
TMT^{10}-128C	D21-DFBS-1	D3-SFM-1	D21-DFBS-2	D21-DM-2	D7-DFBS-3	D21-SFM-3
TMT^{10}-129N	D3-DFBS-3	D21-DM-3	D7-DM-3	D14-SFM-3	D14-DFBS-4	D7-SFM-2
TMT^{10}-129C	D7-DM-2	D1-undiff-5	D3-DM-4	D3-DFBS-2	D1-undiff-4	D21-DFBS-4
TMT^{10}-130N	D21-SFM-2	D21-DFBS-3	D14-DFBS-3	D1-undiff-1	D14-DM-2	D14-SFM-1
TMT^{10}-130C	D14-DM-3	D3-DFBS-4	D21-SFM-1	D7-DM-4	D21-DM-1	D7-DFBS-2
TMT^{10}-131N	D21-DM-4	D14-SFM-4	D7-DFBS-4	D1-undiff-6	D3-SFM-3	D3-DM-2
TMT^{11}-131C	Pool	Pool	Pool	Pool	Pool	Pool

Supplementary Figures

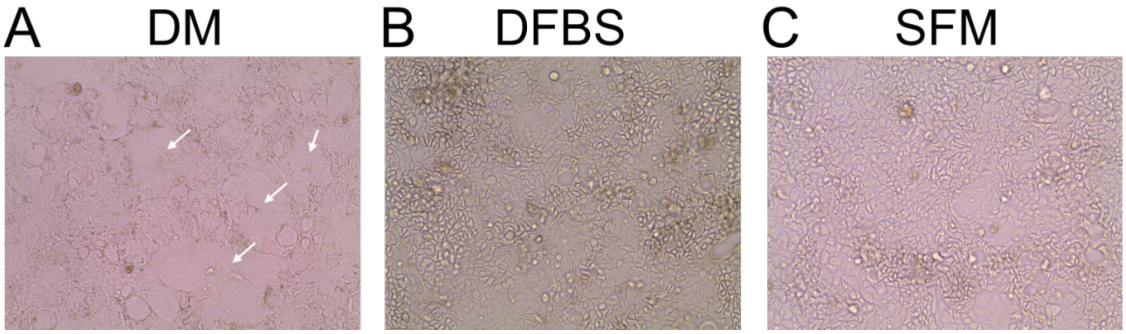

Supplemental Figure 1. Microscopic images comparing cell morphology during the differentiation of Caco-2 cells. EVOS bright-field image of Caco-2 cells cultured for 7 days in DM (A), DFBS (B), SFM (C). White arrows indicate dome-like structure, an indicator of intestinal cellular differentiation.

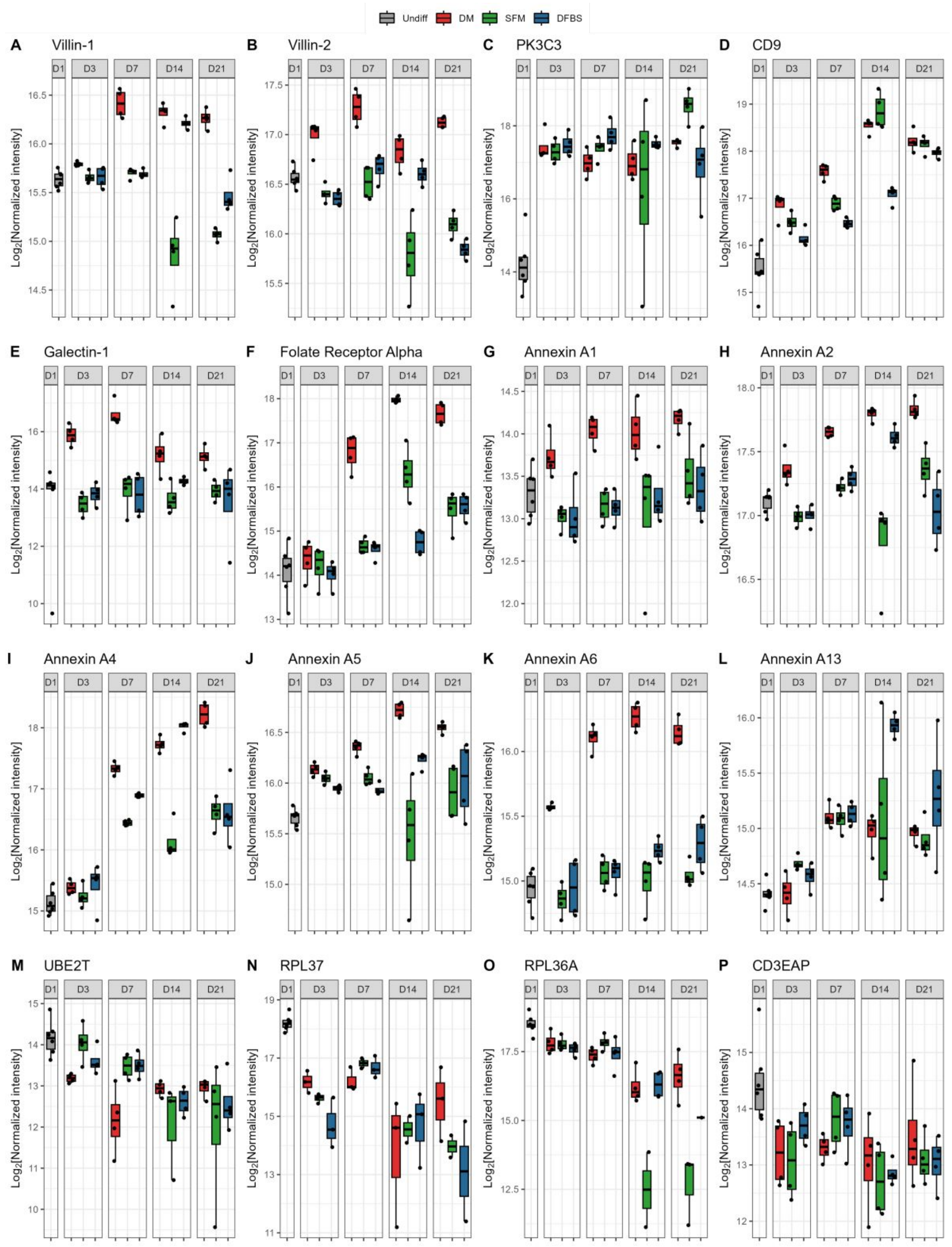

Supplemental Figure 2. Differential protein expression of selected proteins in key Caco-2 cell differentiation groups. Log$_2$(Normalized Intensity) of Villin-1 (A), Villin-2 (B), PK3C3 (C), CD9 (D), Galectin-1 (E), Folate Receptor Alpha (F), Annexin A1 (G), Annexin A2 (H), Annexin A4 (I), Annexin A5 (J), Annexin A6 (K), Annexin A13 (L), UBE2T (M), RPL37 (N), RPL36A (O), CD3EAP (P) over time.

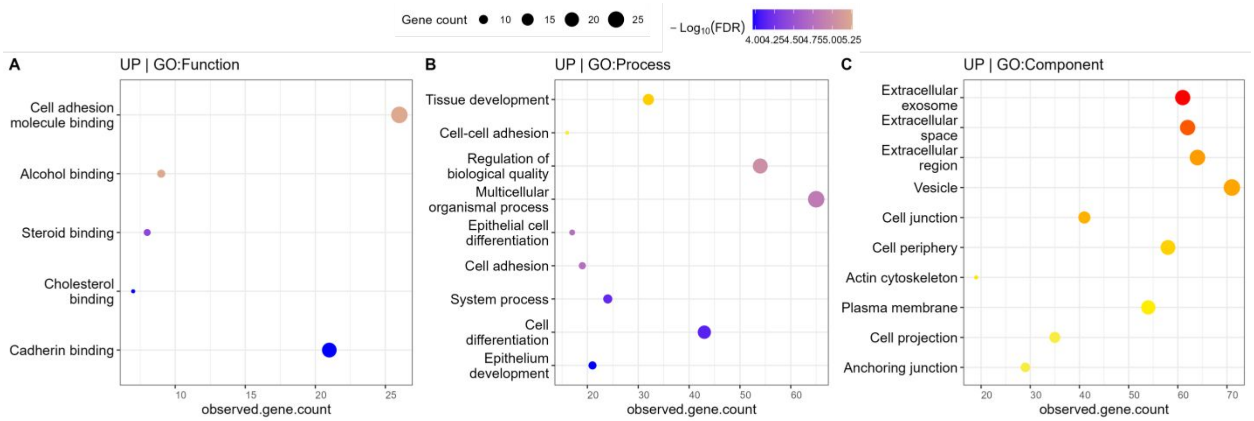

Supplemental Figure 3. Functional enrichment analysis of proteins collected from differentiated and undifferentiated Caco-2 cells which were further upregulated in the D7DM group as compared to D21DFBS and D21SFM groups. Gene Ontology enrichment analysis using STRING of PLSDA VIP ≥ 1 proteins up-regulated functions (A), processes (B), and components (C).

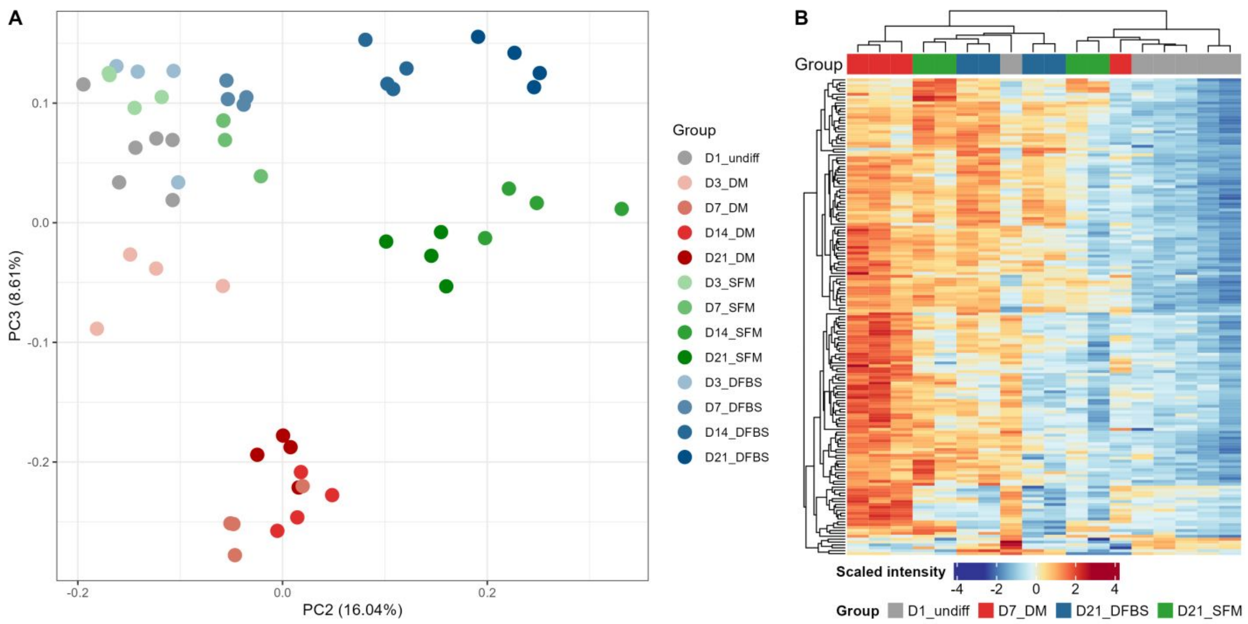

Supplemental Figure 4. Overall trends in N-glycosylated proteins of Caco-2 differentiation from carcinoma into intestinal epithelial-like cells. PCA analysis (A). Heatmap and clustering of all Q100 N-glycosylated proteins (B).

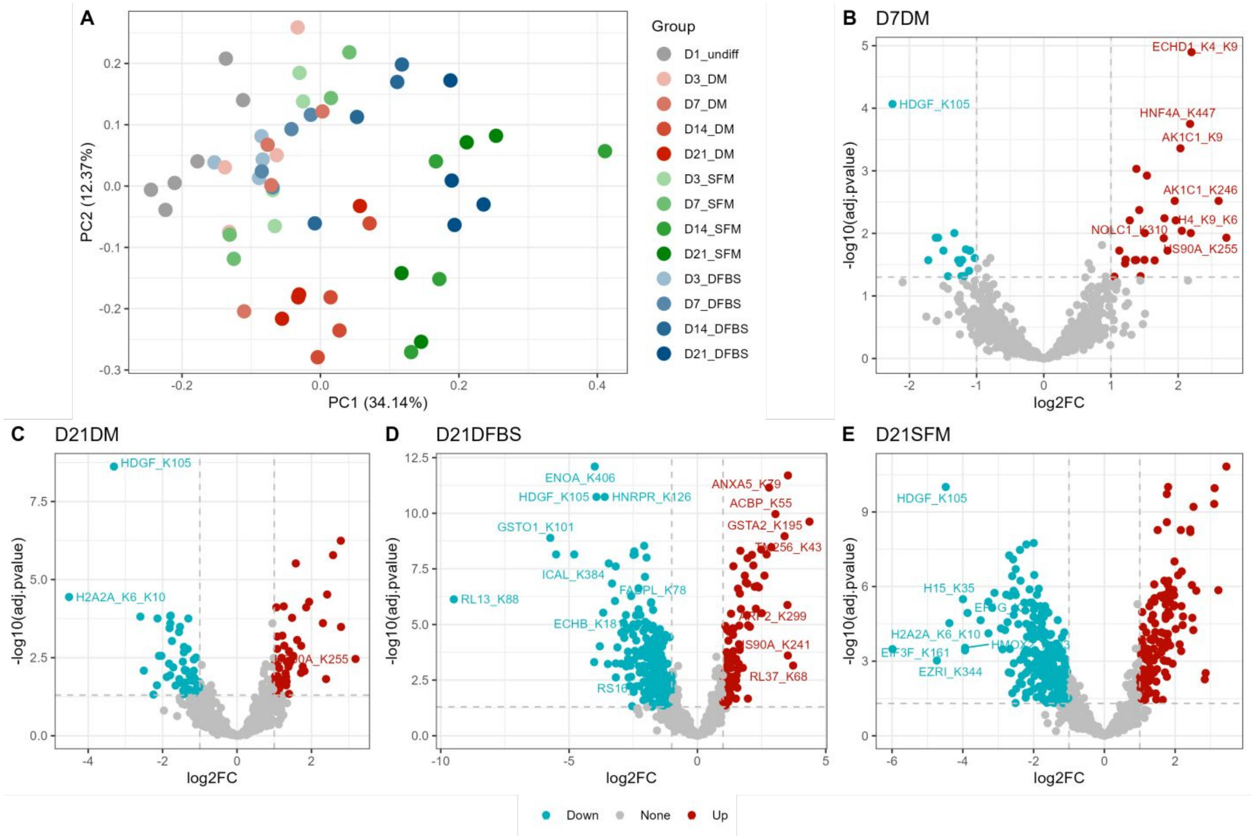

Supplemental Figure 5. Overall trends in acetylated proteins of key differentiated Caco-2 cell groups compared to undifferentiated cells. PCA analysis (A). Volcano plot of Log2FC of all lysine acetylated proteins, highlighting those with a fold change ≥ 2 and adjusted p-value ≤ 0.05 comparing undifferentiated day 1 Caco-2 cells to cells after 7 days of growth in DM (B), or 21 days of growth in DM (C), DFBS (D), and SFM (E).

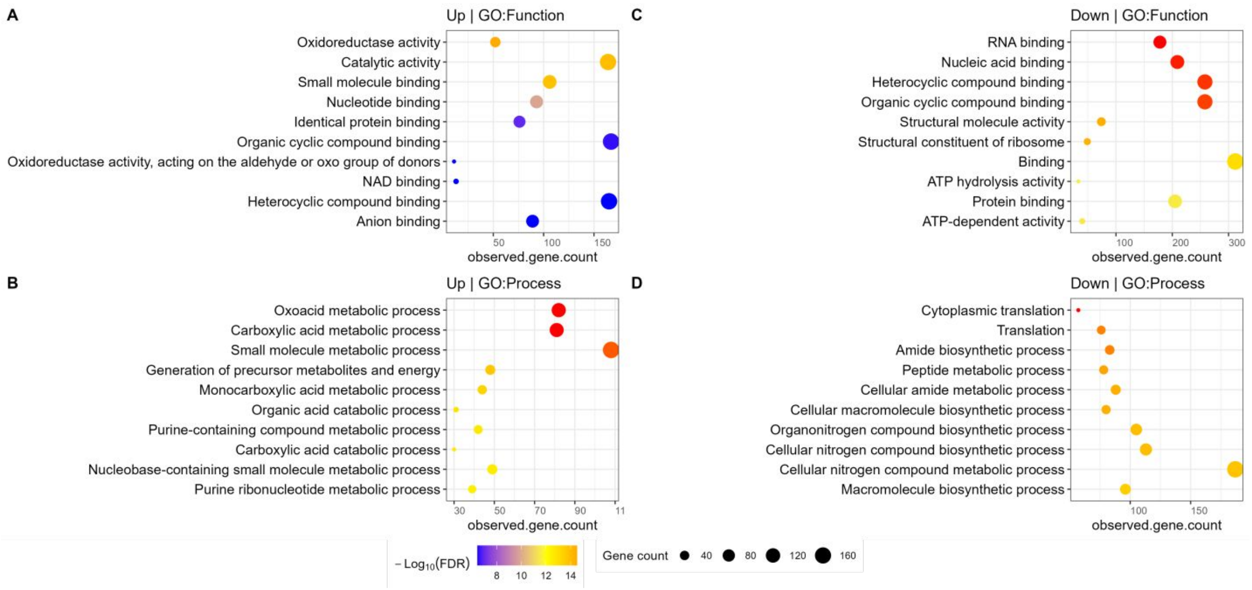

Supplemental Figure 6. Functional enrichment of lysine acetylated proteins in differentiated cells (D7DM, D21DFBS, D21SFM) compared to undifferentiated (D1undiff) cells. Gene Ontology enrichment analysis using STRING of PLSDA VIP ≥ 1 proteins upregulated functions (A) and processes (B); down regulated functions (D) and processes (E).

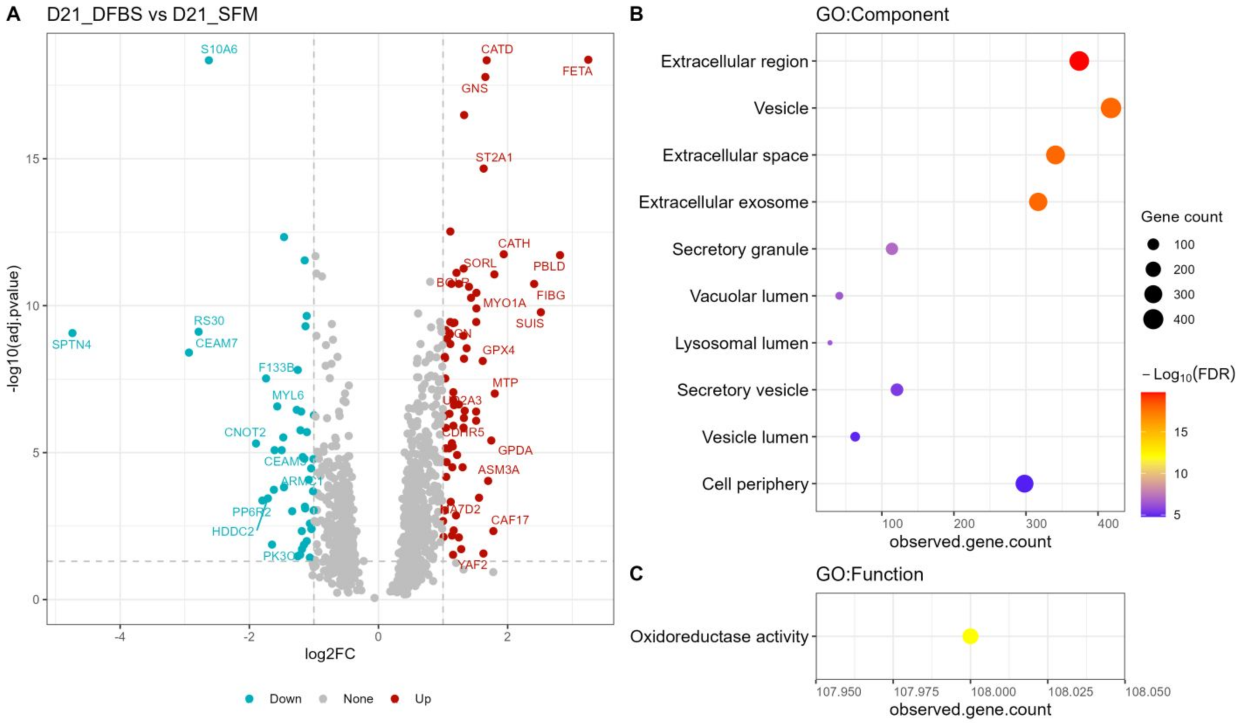

Supplemental Figure 7. Differences between spontaneously differentiated Caco-2 cells cultured using either DFBS or SFM media at day 21. Volcano plot of Log2FC of all proteins, highlighting those with a fold change ≥ 2 and adjusted p-value ≤ 0.05 comparing spontaneously differentiated Caco-2 cells after 21 days of growth in DFBS versus SFM (A). Gene Ontology enrichment analysis using STRING of PLSDA VIP ≥ 1 differentially regulated proteins enriched components (B) and functions (C). No biological process GO term is significantly enriched.
